# Using research networks to generate trustworthy qualitative public health research findings from multiple contexts

**DOI:** 10.1186/s12874-019-0895-5

**Published:** 2020-01-21

**Authors:** Lot Nyirenda, Meghan Bruce Kumar, Sally Theobald, Malabika Sarker, Musonda Simwinga, Moses Kumwenda, Cheryl Johnson, Karin Hatzold, Elizabeth L. Corbett, Euphemia Sibanda, Miriam Taegtmeyer

**Affiliations:** 1 Liverpool School of Tropical Medicine, Liverpool, UK; 2 BRAC James P. Grant School of Public Health, Dhaka, Bangladesh; 3 Institute of Global Health, University of Heidelberg, Heidelberg, Germany; 4 Zambia AIDS Related Tuberculosis Project, Lusaka, Zambia; 5 Malawi Liverpool Wellcome Trust, Blantyre, Malawi; 6 World Health Organization, Geneva, Switzerland; 7 Population Services International, Johannesburg, South Africa; 8 Clinical Research Department, London School of Hygiene and Tropical Medicine, London, UK; 9 Centre for Sexual Health and HIV AIDS Research Zimbabwe, Harare, Zimbabwe; 10 Department of International Public Health, LSTM, Pembroke Place, Liverpool, L3 5QA UK; 11 Tropical Infectious Diseases Unit, Royal Liverpool University Hospital, Prescot Street, Liverpool, L7 8XP UK

**Keywords:** Qualitative research, Research networks, Trustworthiness, Generalisable research, Research guiding principles, Research good practices

## Abstract

**Background:**

Qualitative research networks (QRNs) bring together researchers from diverse contexts working on multi-country studies. The networks may themselves form a consortium or may contribute to a wider research agenda within a consortium with colleagues from other disciplines. The purpose of a QRN is to ensure robust methods and processes that enable comparisons across contexts. Under the Self-Testing Africa (STAR) initiative and the REACHOUT project on community health systems, QRNs were established, bringing together researchers across countries to coordinate multi-country qualitative research and to ensure robust methods and processes allowing comparisons across contexts. QRNs face both practical challenges in facilitating this iterative exchange process across sites and conceptual challenges interpreting findings between contexts. This paper distils key lessons and reflections from both QRN experiences on how to conduct trustworthy qualitative research across different contexts with examples from Bangladesh, Ethiopia, Kenya, Indonesia, Malawi, Mozambique, Zambia and Zimbabwe.

**Methods:**

The process of generating evidence for this paper followed a thematic analysis method: themes initially identified were refined during several rounds of discussions in an iterative process until final themes were agreed upon in a joint learning process.

**Results:**

Four guiding principles emerged from our analysis: a) explicit communication strategies that sustain dialogue and build trust and collective reflexivity; b) translation of contextually embedded concepts; c) setting parameters for contextualizing, and d) supporting empirical and conceptual generalisability. Under each guiding principle, we describe how credibility, dependability, confirmability and transferability can be enhanced and share good practices to be considered by other researchers.

**Conclusions:**

Qualitative research is often context-specific with tools designed to explore local experiences and understandings. Without efforts to synthesise and systematically share findings, common understandings, experiences and lessons are missed. The logistical and conceptual challenges of qualitative research across multiple partners and contexts must be actively managed, including a shared commitment to continuous ‘joint learning’ by partners. Clarity and agreement on concepts and common methods and timelines at an early stage is critical to ensure alignment and focus in intercountry qualitative research and analysis processes. Building good relationships and trust among network participants enhance the quality of qualitative research findings.

## Background

As the push for production of generalizable evidence to inform policy and practice becomes ever greater [1, 2], single country cases or explanatory controlled trials are often viewed as insufficient to influence policy and practice decisions. Global regulatory and normative bodies such as the World Health Organization (WHO) rely on high-quality evidence from different contexts for normative work such as guidelines development and understanding of societal values and preferences [1].

Research networks, or consortia, are growing in popularity as a means of conducting research across contexts [3–6]. Such networks bring together teams from different sites with relevant contextual knowledge, relationships and skills to strengthen and augment the global evidence base [7–9]. These networks often apply multidisciplinary research approaches to implementation research, including a strong central element of social science using qualitative methods. If qualitative research approaches are used in formative research, findings can feed into the design of interventions to ensure the interventions meet the needs of target populations. If used in evaluation, findings provide explanation of what worked, for whom and why, informing sustainability and scale up. Research networks are a valuable means to the democratisation of, and wider participation in, the production of trustworthy evidence, especially when addressing operations or implementation research questions in real life settings or pragmatic trials.

Regardless of the context(s) in which it is applied, qualitative research is fundamentally interactive, collaborative and based on exploring and understanding perceptions and experiences [10, 11]. In addition, the quality of qualitative research data collected depends on the experience of the researcher collecting it as much as the methods and tools used – which brings additional challenges to research teams stretched across geography and times zone. To be trustworthy, qualitative research should be rooted in a strong understanding of the local context, the researchers’ positionality and developed iteratively through multiple rounds of joint discussion [12, 13]. There is a longstanding debate about how to ensure rigour in qualitative analysis without losing its value, which in turn is shaped by the epistemological stance of the researcher or research team [11, 14–17]. Qualitative research networks (QRNs) can face practical, logistical and financial challenges in facilitating a meaningful iterative exchange process alongside conceptual difficulties in interpreting findings across contexts. Despite facing similar public health challenges, research sites may vary considerably in terms of their history, exposure to research including being over- or under- researched, cultural and gender norms, community structures and health systems.

This paper distils key lessons learned from two QRNs on how to conduct trustworthy (high quality) qualitative research across contexts. In qualitative research, trustworthiness is a concept that encompasses several dimensions, which include credibility, dependability, confirmability and transferability [10]. We briefly define these dimensions (quantitative parallels provided in parentheses). *Credibility* (internal validity), is concerned with how congruent the findings are with reality [18]. Approaches to enhance credibility include: prolonged engagement, triangulation, saturation, rapport building, iterative questioning, member checking, inclusive coding approach where all themes are coded iteratively rather than reduced to fit predetermined criteria and reflexivity [10, 19, 20]. *Dependability* (reliability) is the degree to which a study can be replicated, and whether, when there is more than one observer, members of the research team agree about what they see and hear [10, 21]. *Confirmability* (objectivity) is neutrality of researcher in interpreting findings [22]; findings being free from bias, including social-desirability bias, which can be inherent since researchers design and execute tools. Maintaining reflexivity is key to managing such bias. *Reflexivity* is the consideration and acknowledgment of how one’s beliefs and experiences can influence the research process, including participant responses and how data are collected, interpreted, analysed and presented [10]. However, regardless of reflexivity involved, biases cannot be completely ruled out. *Transferability* (generalisability) is applicability of findings to other contexts and achieved through thorough description of study context and assumptions [21]. This is somewhat contentious in qualitative research as it has been argued this may belittle the importance of context [23] and hence is an area of debate in qualitative research with different type of generalisabilility discussed as explained in Table 1 [24, 25].

**Table 1:** Types of generalisation in qualitative research [24, 25]

Type of generalisation	Brief description
Theoretical/conceptual	From local data observations to general level; theory emerging from analysis and interpretation; concepts developed based on data can be applied elsewhere.
Empirical /Analytical	Generalise about and to other social processes in similar or different settings.
Analogical	Generalising from one or more cases to analogous (similar) cases; One or more characteristics in one case may be adaptable to/actionable in other analogous cases. This can be more applicable to case study research
Communicative	Effectively communicate with target audience with adequate contextualisation so the reader can assess study evidence similarity with their own setting.
Naturalistic	Generalisation a function of people’s knowledge based on their experiences; empowers the readers and democratises generalisation; provides sufficient context for reader to judge applicability of study findings to their world.

### Methodology

The development of the ideas herein was a fundamental part of the thinking of each consortium. Two authors (LN for STAR and MBK for REACHOUT) coordinated the day-to-day activities of the QRNs as research managers of the respective consortia and writing the paper occurred within that context. The two consortia frequently conduct face-to-face meetings (at least twice a year for STAR and at least once a year for REACHOUT) with scheduled monthly teleconferences in between the meetings. Therefore, discussing and writing the paper happened in that context of constant interaction among the QRN members. After MT conceptualized the idea for the paper, it was shared and discussed with LN who developed an initial draft that was built on in subsequent stages. At a scheduled REACHOUT face-to-face annual meeting, LN, MBK, ST, MS built on the initial draft to develop a more detailed comparison of the two cases including examples from participating countries. After this meeting, LN and MBK drafted the paper based on the new structure and developed STAR and REACHOUT examples, respectively. To obtain the STAR examples, LN relied on the scheduled monthly teleconferences and bi-annual meetings, which he coordinated. Likewise, MBK relied on scheduled teleconferences and annual meetings as well as ongoing positionality discussions to obtain REACHOUT examples. QRN members beyond the authors commented on the identified key messages and suggested improvements, thereby providing a somewhat removed/distant critique to the theme generation process, which enriched the theme refinement process. After each stage of paper draft development, LN and MBK worked with ST and MT (some of the senior colleagues in the two consortia) for more pointed and strategic guidance. Therefore, the process of generating evidence for this paper, including guiding principles and good practices, followed a thematic analysis method: themes initially identified were refined in an iterative process until final themes were agreed upon in a joint learning process. At every stage of refining the paper, minutes were kept with action points for authors followed up by coordinators of the two QRNs. Studies conducted under both STAR and REACHOUT were approved by ethics boards in participating countries and institutions.

### Description of the QRNs: self-testing in Africa (STAR) and REACHOUT

The QRN is the new method the paper is suggesting as an approach to generating trustworthy qualitative public health research findings from multiple contexts. In this section, we present the projects within which the two QRNs operated and compare and contrast the QRNs. The Self-Testing in Africa QRN (hereafter referred to as STAR-QRN) is a part of a wider network of researchers and implementers working in Malawi, Zambia and Zimbabwe [26]. Members of the STAR-QRN are drawn from various disciplinary backgrounds including social sciences, medicine, marketing and epidemiology. The STAR consortium sought to investigate how best to reach people with HIV self-testing (HIVST) services in an effective, efficient and ethical manner, and how to link those testing to healthcare. This is a multi-method, multi-level and multi-country study involving clinical performance studies, randomised control trials, discrete choice experiments, household surveys and qualitative studies. As part of this wider research consortium, the STAR-QRN tackled questions including preferred distribution models of HIVST kits, optimizing performance of self-testers and social harms related to HIVST. QRN findings directly influenced HIVST distribution, distribution model development and refinement, and was used in process evaluation to understand/explain findings.

The REACHOUT consortium is a QRN that seeks to understand and improve the quality of care from close-to-community providers of health care in Malawi, Mozambique, Kenya, Ethiopia, Bangladesh and Indonesia [7]. Qualitative methods have been used to explore core areas including: motivation, supervision, behaviour, attitudes, quality improvement and practices. Beyond research, the programme is focusing on implementation of supportive supervision and quality improvement capacity development at multiple levels of the health systems. In Table 2, we provide a succinct comparison of the two QRNs across domains of interest.

**Table 2:** Comparing the QRNs

Domain	STAR	REACHOUT
Disciplinary focus	Both are interdisciplinary involving social science, anthropology, economics and health systems, and other stakeholders including policymakers and those involved with implementing interventions	
Health area scope and focus	Focused on HIV self-testing, introduction of a new approach and technology; increasing coverage is priority.	Flexibility on topic of focus (maternal, neonatal and child health, tuberculosis, abortion) and cross cutting issues (e.g. motivation, supervision, quality improvement), under the umbrella of close-to-community provision; improving quality is priority.
Contexts involved	More homogeneous contexts of South/East Africa and a common implementer in Population Services International (PSI) marketing strategy and training curricula.	Works with multiple actors (government, NGO etc.) and across a wider geography with both African and Asian partners and contexts.
Role of the QRN in the consortium	Overall study design is a series of cluster randomised trials informed and explained by the QRN’s work.	Holistic health systems framing driven by the QRN.
Non-researchers in the QRN	Both have many actors (such as policy makers; HIVST distributors; frontline health workers, and clients) to interact with during research process.	
Meeting modality	Both conduct regular periodic face-to-face consortium meetings and teleconferences to allow for exchanges and to facilitate analytical discussion across different contexts.	

## Results

### Principles and good practices for conducting trustworthy research in QRNs

**Fig. 1:**
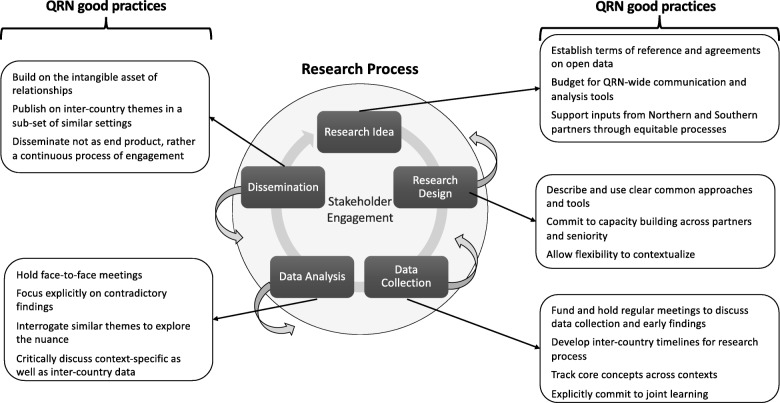
Good practices for QRNs mapped onto the research process

We propose four cross-cutting principles (italics and numbered list below) to underpin trustworthy qualitative public health research that spans multiple contexts. We describe each principle and demonstrate how it was applied, illustrated by concrete examples and summarised into good practices specific to each phase of the research process, as shown in Fig. 1.

#### Principle 1: Be open. Use explicit communication strategies that sustain dialogue, build trust and encourage collective reflexivity

Good communication through an open dialogue approach allows QRN participants to critically discuss data and interrogate different interpretations of findings. Collective agreement on approaches to dialogue is important as working across sites creates communication barriers such as language, time zone differences, and connectivity problems. First, there is no substitute for face-to-face meetings to build relationships and trust and this needs to be appropriately budgeted. Second, in both networks having a range of modalities (including monthly calls, regular email exchanges, application messaging reminders and online file sharing) supported effective communication.

Without trust among QRN participants cultivated over time, technical solutions to communication challenges would have been insufficient. Trust reduces complexity and helps decision making to be based on experience while using past knowledge to reduce risk [27]. The face-to-face meetings set the tone and values for the two consortia and such meetings were key to creating safe spaces for open and honest discussion of the data and interpretation of the results from multiple perspectives. Early in both STAR and REACHOUT projects, to facilitate trust and collaboration, we established agreements on open sharing of data and other resources. We also developed a data management protocol, which provided guidelines such as the requirement to ensure that there were no identifiers in the public domain. One of the authors (MT) was the principal investigator for REACHOUT and led the STAR QRN within the STAR Consortium, which also contributed to cross learning between the two projects in terms of data sharing.

We enhanced credibility, dependability and confirmability by encouraging intercountry and interdisciplinary discussions, which prompted country team members to analyse data through a wide range of disciplinary and professional lenses. We used the face-to-face meetings and webinars as capacity building opportunities to reduce the ‘distance’ between perspectives, encompassing short trainings and exchanges around qualitative research methods and measures to ensure trustworthiness. Furthermore, QRN members suggested areas requiring more probing, which enriched the data collection and analysis process. Thus, the QRN allowed for triangulation of perspectives, enabling research team members from different cultural, academic, age and gender backgrounds to input meaningfully into the process and build empirical and conceptual generalisability. For instance, in REACHOUT, using ‘discussion rounds’ to get rapid reflections from each participant during member country meetings constituting participants of varied levels of seniority or using anonymous post-it contributions to discussion topics were a couple of ways that we tried to overcome hierarchy and power while acknowledging the variety in perspectives. Discussion rounds allowed all members to express their views and the anonymous post-in notes gave further room to those who might have been reluctant to speak in presence of seniors or peers. However, such a process can not be said to have completely overcome hierarchy; participants were from different cultural backgrounds where expression of opinion in presence of superiors can be viewed differently. Therefore, deliberate efforts and mechanisms to sustain an atmosphere of openness is something the QRN encouraged and suggest to be an area other researchers should pay attention to.The QRN enabled collective reflexivity, which ensured that there were no “untrammeled incursion of values in the research process” [10]. At the beginning of the project in the STAR QRN, the three countries involved in STAR phase 1 (Malawi, Zambia and Zimbabwe) were differentially exposed to HIVST. As such, some attitudes towards self-testing had already formed among some members. Members were encouraged to continuously reflect on such attitudes and their (potential) impact on the research process.

The continued engagement enabled members to note when personal, national or disciplinary values were improperly influencing the research process or impeding openings to new learning. For instance, in face-to-face STAR QRN meetings, QRN members discussed how a biomedical definition of social harms related to HIVST would hinder the ground-up definition and interpretation of social harms and benefits by those experiencing the phenomena. The members observed that the biomedical definition was not informed by input from people experiencing social harms or with potential to experience the harms. As such, we removed the word adverse events or serious adverse events, which are predominantly used in biomedical research in relation to harms and instead used social harms and serious social harms, terms that HIVST clients would easily relate to (Kumwenda et al 2018, submitted). QRN members further noted that such definitions and interpretations of harms should not be infinitely open-ended as to render them overly complex and of less public health value. As a result, we developed guidelines on capturing social harms from the perspectives of people experiencing or with the potential to experience harms.

##### QRN good practices

- Build on the relationships within and beyond the research team to strengthen joint analysis and broaden dissemination beyond publications.
- Commitment to capacity building to enable broad contribution to analysis and publication across partners and levels of seniority.
- Joint learning (drawing lessons from the implementation process) as an explicit commitment of partners in the QRN.
- Equitable processes to support inputs from Northern and Southern partners (e.g. in Project Management committee).

#### Principle 2: Be flexible. Jointly develop approaches to data collection, sharing and analysis

From the outset, agreements on methodological approaches (including level of flexibility), data collection and sharing tools, and the overarching research goals were collectively discussed and agreed in face-to-face meetings of both QRNs. Key items shared between partners in the research design and data collection included: methods manuals, interview topic guides, field notes, training and instructions for use in relation to any intervention, and relevant literature. Teams had flexibility to add additional questions or respondents of interest to expand on the common core approach.

In the analysis phase, in REACHOUT, all but one (Bangladesh that use Atlas Ti) of the network members used the same data handling software (QSR NVivo 10) to facilitate collective data handling and management. Framework analysis, which has a mixture of deductive and inductive approaches [25, 28], was selected as a common analytical approach. The framework method has clear steps to follow, making it appropriate for multi-disciplinary teams where some members have less experience in qualitative research. For instance, in the STAR-QRN, one topic in all countries involved users’ preferences in relation to HIVST. Through the joint analysis exercises, we found that there were more similarities in preferences for HIVST between in-school youth in all contexts than between adolescents’ experiences in rural and urban settings in any one context [29] thus, highlighting empirical generalizability in some but not all aspects of the findings. In REACHOUT, we observed that when the identifiers were removed from quotes on maternal health practices in Indonesia and Ethiopia, the data collection teams themselves were unable to identify which context they came from, highlighting similarities in findings across two very different contexts.

In both QRNs depth of analysis was enhanced and enriched through iterative conversations; with those leading the collection and management of such data. In STAR QRN, discussion/sharing led to formulation of a common coding framework that was used across countries. In REACHOUT, teams shared coded datasets with other network members with the understanding that this common intellectual property would elicit discussions on how to disseminate and publish. Using common data management guidelines offered guidance on how transcripts should be named, how emerging themes should be handled, and when to explicitly look at variations between respondent types. These measures helped support comparative analysis and were meant to provide focus rather than restrict members, allowing for open dialogue and flexibility as well as credibility and dependability of the findings.

While qualitative data analysis is an ongoing process, there are phases in the research process where data analysis is more intense and focused. One such phase is when researchers pay more attention to the transcripts during the initial coding and interpretation of the data. For example, in face-to-face meetings of STAR-QRN during this phase, we carried out joint analysis exercises including development of intercountry coding frameworks based on research questions and objectives (deductively developed) and informed by study findings (inductively). To inform the coding framework developed deductively, members familiarised themselves with interviews and conducted joint coding. The joint coding sessions involved members sharing transcripts purposefully selected from the three countries to gauge common understanding of the themes. Data from the transcripts were not de-identified because the projects already set out to do intercountry analysis where project members were permited to access de-identified data. Building on this common understanding, country research teams proceeded with the rest of the coding and continued to communicate. Such a process enhanced credibility, dependability and confirmability of study findings.

In both QRNs, flexibility was required in setting timelines for field work and analysis. Related to the realities of ethical approval timelines and processes, inter-country analysis did not follow immediately from in-country analysis in all sites. However, such delays sometimes allowed for further reflection and discussion that informed in-country research process for teams still in the field. Such sustained reflection and discussion enhanced confirmability and credibility in that research teams related their findings at intra and intercountry levels. In STAR QRN, there was a tension between the urgency from marketing teams and desire to quickly show impact and ensuring research properly informed distribution of self-test kits, a process helped by the involvement of implementers (HIVST kit distributors).

##### QRN good practices

- Establish terms of reference and agreements on open data
- Share clear common approaches and tools (e.g. methods manuals and joint analysis approach)
- Collectively conduct data analysis on selected inter-country questions according to agreed approach
- Allow flexibility and sustained dialogue to contextualize findings, within parameters
- Develop inter-country timelines for research process with sufficient cushioning
- Budgeting for QRN-wide analysis and communication tools

#### Principle 3: seek common understanding. Facilitate exchange through dialogue, visits to each other’s sites, translation of contextually embedded concepts

We used backtranslation and exchange field visits to enhance common understanding of research findings. With regards backtranslation, we noted that using the same word in the common language (English) of the QRN did not always generate a common understanding. This is true even in the literature, where “community health worker” has been defined, categorized, redefined and expanded to cover different cadres [30, 31] – and this is exacerbated by translation. In STAR-QRN, we developed a table where the key words and concepts were back translated. Back translation involves translating a word or concept from one language (such as English) into a local language for the purpose of fieldwork, then translating that translation back to the original language to give voice to the nuance and elicit discussion on how that influenced respondents (or not) in different country contexts [32]. Such a process allowed for credibility, dependability as well as empirical and conceptual generalisation of study findings.

Another approach to building common understanding that generated interesting discussions about empirical and conceptual transferability was exchange visits. In REACHOUT, the face-to-face meetings were hosted by each participant country over the lifetime of the QRN (Table 3). As a core component of each of these meetings, field site visits and exposure to close-to-community (CTC) providers in the country supported understanding across very different contexts (for example the informal settlements in Dhaka, Bangladesh and rural Manhiça in Mozambique) – and inspired innovation and change in the home health system, documented in blogs and personal reflections [7].

**Table 3:** Use of South-South exchange visits to strengthen shared understanding in REACHOUT

As part of strengthening cross-contextual understanding, in the REACHOUT QRN face-to-face meetings were organized to rotate through all the participating network sites over the lifetime of the project. This meant, in parallel with joint research approach and tool development, analysis, and capacity building, all team members were able to make field visits to project sites in other countries. Learning about the health system through direct observation and conversation with providers and users made deep impressions and led to more productive analysis discussions. We also involved national and district policymakers working with the project on field visits to other countries, sharing ideas and experiences for both relationship deepening and joint learning, explicit goals of the project.	

##### QRN good practices

- Have a tool to track core concepts across languages and contexts (e.g. back translation table)
- Face-to-face meetings to progress discussion of results and positionality
- Regular meetings to discuss data collection and early findings
- Dedicated funding to support above activities

#### Principle 4: embrace complexity. Support empirical, conceptual and analogical transferability

In both STAR-QRN and REACHOUT, sustained conversations about perspectives and positionality gave a common lens through which data from different countries could be analysed and interpreted. This allowed complexity of different identities and different settings to remain while working toward a common understanding. Intercountry analysis was aimed at identifying areas of thematic convergence while highlighting areas of difference. For example, in two countries where governance of the healthcare system had been devolved (Kenya and Indonesia) on different time scales, comparing data from the two sites allowed us to look at the influence of context under similar health system governance, whereas comparing data from those two sites to the others gave an idea of the influence of devolution on the intervention [33]. Throughout the analysis process, we looked for commonalities to see where similar contexts yielded similar results and where they differed, viewing both types of findings as valuable. Not all inter-country findings were generalized across all settings; some publications were limited to a sub-set [33–37]. A detailed example of empirical and conceptual transferability is captured in Table 4.

**Table 4:** Empirical and conceptual transferability of findings on social harms related to HIVST

In the STAR QRN, one of the themes we set out to explore and describe in the three countries was that of social harms in relation to HIVST. Forced testing was an example of such harm. In Zimbabwe, respondents in a focus group with community members discussing the social harm of forced testing wondered why and how forced testing was bad. They asked focus group facilitators to explain why it was bad to force one’s child, spouse or relation, explaining that it was for the good of those being forced to test because that would lead to accessing proper care and treatment. It should be emphasized that these people did not actually force others to test; it was only an attitude or perception that they had. Such understanding is something we did not anticipate, and we called the phenomenon “compassionate-forced testing” (CFT) since the forced testing was done out of perceived compassion for the one being ‘forced’. Still in Zimbabwe, some respondents argued that some people, such as house servants who look after children, must be forced to test to protect the children. Although no reports of actual forced testing emerged, we termed this precautionary-forced testing (PFT) since the intention of the intended forced testing was to act as a precautionary measure to protect the children being looked after. Other terms that emerged in relation to the actions taken by people to make others test included ‘persuade’, and ‘convince’, which were less intrusive. Such concepts emerged inductively from the data, were common across contexts and had an agreed definition within the STAR QRN, allowing them to be incorporated into the common coding framework. CFT was empirically and conceptually applicable in Malawi among couples and in Zambia among families where some parents applied it to their children. PFT was empirically and conceptually transferable among married women in Malawi and youths in Zambia who reported the acceptability of PFT directed at their partners, albeit with the intention of being direct beneficiaries of the intended prevention rather than children as was the case in Zimbabwe. As was the case in Zimbabwe, CFT and PFT in Zambia and Malawi were based only on people’s attitudes and perceptions; actual forced testing did not occur. In the second phase of STAR initiative, we have employed community-led models of HIVST where communities decide on how, who, where and when HIVST should be delivered. Such community-led initiatives are some of the mesures to enhance sensitization around the need to ensure people take HIVST following informed consent.	

One means of managing complexity was collaborating with the users of the research throughout the network process to continually (re-)focus on their priorities. In STAR-QRN, involving implementers who marketed and distributed HIVST kits enriched the research process by informing research questions and probes, and the implementers acquired real time feedback from the research process to improve marketing strategies and distribution models. Furthermore, development of a training curriculum for distributors of HIVST kits was informed by formative research that pointed to areas that needed more attention during the training. In so doing, the real-time feedback among researchers and implementers was critical in generating evidence to inform guidelines, such as those put forward by WHO, for rolling out and scaling up of HIVST. In REACHOUT, engaging Ministries of Health officials, particularly community health departments, ensured that the questions were aligned with the broader health system goals and policy initiatives, and that the quality improvement interventions among CTC providers had input and engagement of key government ministries and stakeholders. Such involvement was key to ensuring sustainability of interventions and prioritizing within complexity.

Complexity theory was used as an important framework in the REACHOUT project, where we acknowledged that each healthcare system in which we worked was a complex adaptive system [38]. When researchers generalise across settings, they are sometimes accused of over-simplifying context. Rather than trying to ignore or reduce the complexity of working across environments in the QRN, we developed an understanding of each healthcare system as dynamic and how it might respond to external stimuli and the potential for feedback loops. This gave us a theoretical means of mapping and finding commonalities in the complexity, as we worked to compare actively changing systems.

##### QRN good practices

- Explicit focus on contradictory findings and what they illuminate about specific contexts
- Interrogate similar themes to explore the nuance within and across different contexts and participant groups
- Provide forum for critical discussion of context-specific as well as inter-country data
- Publish on inter-country themes in a sub-set of similar settings (as well as all or one)
- Dissemination not as end product but a continuous process of engagement with stakeholders

## Discussion

Our analysis has suggested how QRNs can generate trustworthy findings by describing how credibility, dependability, confirmability and transferability--- dimensions of trustworthiness--- can be enhanced. We have identified guiding principles including openness, flexibility, seeking common understanding and embracing complexity; principles on how QRNs can be used to generate such findings. We have also related these principles to practical ideas for building the everyday operations in QRNs.

We note, as urged by other scholars [11, 23, 24], that qualitative researchers should not aim at mimicking criteria for ensuring rigour employed in quantitative research because of the epistemological and ontological bases and assumptions underpinning qualitative research. Instead, qualitative researchers should embrace approaches, such as those described in this article, that can be of meaningful utility in qualitative research. While trustworthiness dimensions discussed here are well known in qualitative research literature [10, 22, 23], what is not clear is how such dimensions can be applied to multi-country qualitative research. Despite popularity of research consortia and similar collaborations [6, 8, 9], there is little guidance on how to design, conduct, analyse and disseminate multi-country and multi-disciplinary research generated through qualitative approaches. While quantitative data from multi-country studies can be easily consolidated and analysed, it is not a straightforward process when qualitative approaches are used. In this article, we have suggested how to pay attention to trustworthiness dimensions in multi-country qualitative research, and have provided guiding principles to conducting such research; principles that can act as anchors of the trustworthiness dimensions based on experience from two QRNs encompassing eight countries. We have also suggested good practices tied to the principles that other researchers can consider.

A major limitation of our analysis is that the two QRNs examined here focus on different research topics, work within different contexts and vary with respect to the relative prominence of the QRN in the wider research project. However, we view the differences as highlighting the generalisability of the key principles and good practices, using similarities to underpin the generation of these common lessons.

## Conclusion

Through presenting two QRN cases, we have suggested principles and good practices for how to use research networks to generate trustworthy findings in qualitative public health research that spans different contexts. We have gone beyond describing how analytical approaches can be applied to inter-country analysis of qualitative data and discussed how working through such networks can enhance trustworthiness of the whole research process, from design to policy influence.

Qualitative research is often context-specific with tools designed to explore local experiences and understandings. Without efforts to synthesise and systematically share findings, common understandings, experiences and lessons are missed. The logistical and conceptual challenges of qualitative research across multiple partners and contexts must be actively managed. This should include a shared commitment to ‘joint learning’ throughout the process by all partners. Clarity and agreement on concepts and common methods and timelines at an early stage is critical to ensure alignment and focus in intercountry qualitative research and analysis processes. Building good relationships and trust among network participants enhance the quality of qualitative research findings.

The strengths of QRNs lie in the multiple perspectives and contextual experiences of researchers and other stakeholders involved. Strategies and processes to make these explicit in a learning environment are important in building the value and relevance of QRN to meet public health challenges, particularly for implementation and operations research and perhaps more widely.

## Data Availability

Not applicable.

## Abbreviations

CTC: Close-to-community
HIVST: HIV Self-Test
QRN: Qualitative Research Network
STAR: Self-Testing in Africa
WHO: World Health Organisation
